# The Surgical Management of Intractable Vertigo and Tinnitus Caused by Neurovascular Compression of the Auditory Vestibular Nerve: A Report of Two Cases and Review of the Literature

**DOI:** 10.7759/cureus.86940

**Published:** 2025-06-28

**Authors:** Naoya Shimomura, Naoki Otani, Sodai Yoshimura, Hiroumi Matsuzaki, Ryo Takagi, Koichiro Sumi, Katsunori Shijyo, Atsuo Yoshino

**Affiliations:** 1 Division of Neurosurgery, Department of Neurological Surgery, Nihon University School of Medicine, Tokyo, JPN; 2 Department of Otolaryngology, Nihon University Hospital, Tokyo, JPN; 3 Department of Radiology, Nihon University Hospital, Tokyo, JPN

**Keywords:** auditory vestibular nerve, microvascular decompression (mvd), neurovascular compression, tinnitus, vertigo

## Abstract

Neurovascular compression (NVC) of the auditory vestibular nerve (AVN) may be one of the causes of intractable dizziness and characteristic tinnitus, which are often described as typewriter-like tinnitus. While these symptoms can be alleviated by oral administration of carbamazepine (CBZ), no standard diagnostic and surgical indications and treatment methods have been established. Microvascular decompression (MVD) is effective for these disorders. We describe two cases where MVD resulted in favorable clinical outcomes, and we also review the relevant literature. Our experience suggests that MVD may be an effective treatment option for patients who exhibit a positive response to CBZ, show clear radiological evidence of NVC, and present with both tinnitus and vertigo.

## Introduction

Neurovascular compression (NVC) of the auditory vestibular nerve (AVN) is considered a possible cause of intractable dizziness and tinnitus [1,2]. The main symptoms of these disorders are severe tinnitus that can be described as “typewriter-like” and frequent vertigo [3-6]. Despite the growing recognition of this condition, standardized diagnostic criteria and treatment algorithms remain lacking. In this context, microvascular decompression (MVD) has been applied, though surgical indications are less clearly defined than for other cranial nerve compression syndromes such as trigeminal neuralgia or hemifacial spasm [4-10]. We report two cases of AVN compression with favorable outcomes following MVD. Both cases met the criteria involving a specific clinical triad: responsiveness to carbamazepine (CBZ), radiological evidence of vascular compression in the internal acoustic canal (IAC), and the presence of both vertigo and characteristic tinnitus. We also engage in a review of the relevant literature and discuss surgical strategies. These cases provide further insights into the diagnostic and therapeutic approach to this uncommon but disabling condition.

## Case presentation

Case 1

**Figure 1:**
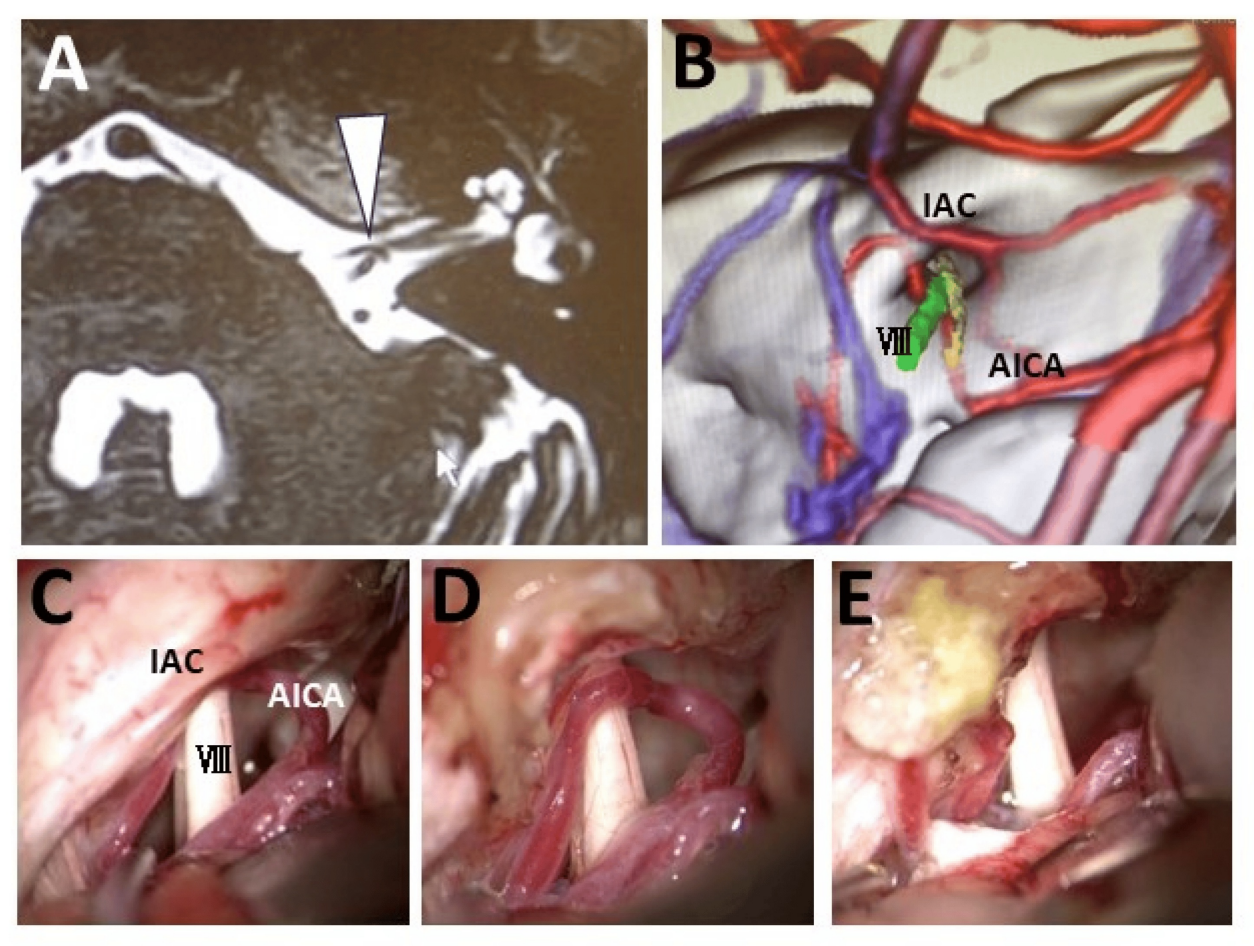
Preoperative imaging and intraoperative field images of Case 1 A: Preoperative head CISS MRI showing the left elongated AICA forming a loop within the IAC and compressing the vestibular nerve (VIII) at the internal acoustic meatus (white arrowhead). B: Fusion image of 3D CT angiography and head MRI showing the AICA compressing the vestibular nerve. C–E: Intraoperative findings confirming cranial nerve compression by the AICA after opening the internal acoustic meatus (C and D), and hence transposition of the AICA was performed (E) AICA: anterior inferior cerebellar artery; CISS: constructive interference in steady state; CT: computed tomography; IAC: internal acoustic canal; MRI: magnetic resonance imaging

The patient was a 58-year-old male who had suffered from progressive vertigo accompanied by left pulsatile tinnitus lasting approximately 10-30 seconds for the past six years. An MRI performed at a local internal medicine clinic had detected no abnormalities. He had been diagnosed with neuromodulated syncope and was treated conservatively. However, his symptoms had worsened, and hence, he had been referred to our department with suspicion of NVC of the AVN. The auditory brainstem response (ABR) was within the normal range. The caloric test showed decreased left-sided response. Constructive interference in steady state (CISS) MRI and 3D CT angiography-venography revealed a vascular loop of the anterior inferior cerebellar artery (AICA) extending into the internal auditory meatus (IAM) and compressing the vestibular portion of the eighth cranial nerve (Figures 1A, 1B). The symptoms improved with oral administration of CBZ. However, the symptoms did not completely disappear despite increasing the medication to the maximum dosage. Therefore, MVD of the eighth cranial nerve was performed. The intraoperative findings corresponded precisely with the preoperative imaging (Figures 1C, 1D), further validating the surgical plan, and transposition of the AICA was performed (Figure 1E). Intraoperative ABR monitoring showed no abnormalities. The postoperative course was uneventful, and the symptoms completely disappeared. No recurrence has been observed in the subsequent three years.

Case 2

**Figure 2:**
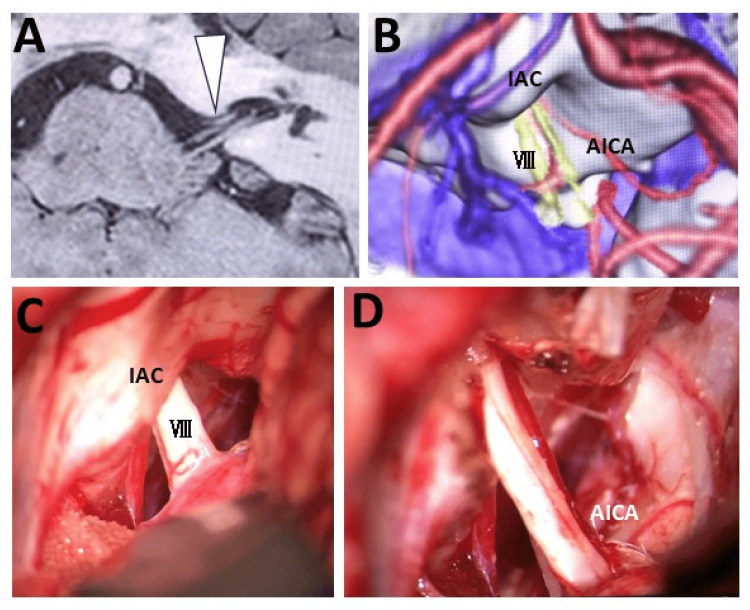
Preoperative imaging and intraoperative field images of Case 2 A: Preoperative head CISS MRI showing the left elongated AICA forming a loop within the IAC and compressing the vestibular nerve (VIII) at the internal acoustic meatus (white arrowhead). B: Fusion image with 3D CT angiography-venography showing the direction of compression by the offending artery and relationships between the anatomical components. C, D: Intraoperative findings confirming cranial nerve compression by the AICA after opening the internal acoustic meatus; hence, transposition of the AICA was performed AICA: anterior inferior cerebellar artery; CISS: constructive interference in steady state; CT: computed tomography; IAC: internal acoustic canal; MRI: magnetic resonance imaging

The patient was a 46-year-old male who had suffered from progressive left pulsatile tinnitus for the past eight years. Severe vertigo had started to synchronize with the tinnitus for four years. As his symptoms had worsened, he had suffered refractory tinnitus accompanied by severe vertigo dozens of times per day. He had been referred to our department with suspicion of NVC of the AVN. CISS MRI and 3D CT angiography-venography showed compression of the left eighth cranial nerve by the AICA in the IAM (Figures 2A, 2B). The symptoms improved with oral administration of CBZ. However, continued oral medication with CBZ became difficult due to the strong side effects such as nausea and vomiting. Therefore, MVD of the eighth cranial nerve was performed. Intraoperative findings confirmed AICA compression of the eighth nerve after opening the IAM, and hence, transposition of the AICA was performed (Figures 2C, 2D). The strong correlation between imaging and operative findings reinforced the value of high-resolution preoperative imaging in surgical planning. Intraoperative ABR monitoring showed no abnormalities. The postoperative course was uneventful, and the symptoms completely disappeared.

## Discussion

Pathophysiology of symptoms caused by NVC

Cerebral vessels may elongate due to aging or atherosclerosis, or become narrower compared to normal; however, as the volume of the posterior fossa remains unchanged, the vessels gradually curve and meander [11,12]. The neuroprotective effect of the nerve sheath is physically vulnerable at the transposition point between the central myelin and peripheral myelin at the brainstem. Therefore, symptoms may occur if a vessel contacts and compresses the myelin sheath transition point [1]. The location of the vulnerable myelin sheath zone varies, the closest to the root exit zone for the glossopharyngeal and vagus nerves at about 2 mm from the brainstem, for the facial nerve at 3 mm, for the trigeminal nerve at 4 mm, and the AVN has a very wide range of 10 mm [1]. Therefore, NVC may affect even the distal side of the AVN. Neuroimaging findings have also suggested that NVC near the IAC will likely induce symptoms [13]. The offending vessels, which curve and meander, initially contact the cranial nerves, but symptoms will presumably develop as the cranial nerves are gradually compressed and displaced, causing nerve damage at the physically vulnerable myelin transition area. In particular, as the offending vessels elongate into the narrow space at the IAC, the compression pressure on the AVN will become greater.

Effectiveness of MVD

The effectiveness rate of MVD for NVC is 83% for trigeminal neuralgia, 91% for hemifacial spasm, and 92% for glossopharyngeal neuralgia [7,8]. On the other hand, the effectiveness rate is lower compared with trigeminal neuralgia and hemifacial spasm: improvement in symptoms of tinnitus was 28%, that in vertigo was 32%, and that in tinnitus and vertigo was 62% [4,5]. The lower effectiveness rate of MVD for AVN reflects the uncertainty of the surgical indication, including MVD's ineffectiveness in cases such as those refractory to CBZ oral administration and/or those with no offending artery on the neuroimaging findings. This ineffectiveness might be affected by long disease duration. Even if MVD is performed more than four years after the onset of symptoms, irreversible neurological damage may have occurred [14]. Therefore, MVD should be performed within the appropriate period, as delays might cause the surgical effect to be weakened due to irreversible damage caused by NVC. Careful patient selection plays an important role in optimizing surgical outcomes. Based on our case reports and a review of the literature, patients who present with both vertigo and characteristic "typewriter-like" tinnitus demonstrate partial improvement with CBZ, and show radiological evidence of neurovascular compression within the IAC, could potentially benefit from MVD.

Surgical strategy and intraoperative monitoring

The compression site by the offending vessels is often located at the peripheral site of the cranial nerve, often within the IAM; hence, opening of the IAC will be necessary to confirm the compression site in the IAM. Both our patients required opening of the IAM to confirm the compression site. Removal of the posterior wall of the IAM by bone drilling requires care to avoid involving the surrounding cranial nerves and cerebral vessels passing through the region. If mastoid air cells have developed up to the posterior wall of the IAM, postoperative cerebrospinal fluid leakage is a risk due to the opening of the mastoid air cells by bone drilling; hence, adequate coating with bone wax and/or fascia is necessary. The AICA will often be the responsible blood vessel. Transposition of the offending AICA requires care that blood flow in its branch, the subarcuate artery, which runs into the IAM, is not obstructed. Full use of the birdlime technique, “a sutureless method that employs a fibrin glue-soaked TachoSil® sheet to secure the transposed vessel in place without damaging surrounding structures,” is necessary to perform such a reliable vascular transposition [15-17].

Complications of MVD for vestibular auditory nerves include hearing dysfunction and brainstem infarction caused by perforator damage. Hearing dysfunction can be caused by tension on the auditory nerve due to brain traction and impaired blood flow in the subarcuate artery during transposition, which can be prevented by continuous intraoperative monitoring of the ABR. We usually perform indocyanine green video-angiography to prevent brainstem infarction caused by perforator damage. However, the perforators behind the surgical field cannot be confirmed. Therefore, motor evoked potential and sensory evoked potential can be useful to confirm the obstruction of these perforators. Intraoperative neurological monitoring is essential for safely completing MVD under these conditions.

## Conclusions

Although no standard surgical treatment has been established for AVN compression, our cases suggest that MVD can be effective in selected patients. Favorable outcomes may be achieved when specific criteria are met, namely, symptom relief with CBZ, radiological evidence of NVC within the IAC, and the presence of both vertigo and characteristic tinnitus. Safe and reliable surgical outcomes depend on appropriate patient selection and meticulous techniques, including decompression within the IAC and intraoperative monitoring. Further case accumulation is necessary to refine indications and improve long-term results.
